# Exploring the impact of public health teams on alcohol premises licensing in England and Scotland (ExILEnS): procotol for a mixed methods natural experiment evaluation

**DOI:** 10.1186/s12874-018-0573-z

**Published:** 2018-11-06

**Authors:** Niamh Fitzgerald, Matt Egan, Frank de Vocht, Colin Angus, James Nicholls, Niamh Shortt, Tim Nichols, Nason Maani Hessari, Cheryl McQuire, Richard Purves, Nathan Critchlow, Andrea Mohan, Laura Mahon, Colin Sumpter, Linda Bauld

**Affiliations:** 1 Institute of Social Marketing, UK Centre for Tobacco & Alcohol Studies, University of Stirling, Stirling, FK9 4LA UK; 2 Department of Public Health, Environments and Society, London School of Hygiene and Tropical Medicine, London, UK; 3 Population Health Sciences, Bristol Medical School, University of Bristol, Bristol, UK; 4 School of Health and Related Research, University of Sheffield, Sheffield, UK; 5 Alcohol Research UK, London, UK; 6 School of Geosciences, University of Edinburgh, Edinburgh, UK; 7 formerly Brighton & Hove City Council, Brighton, UK; 8 Department of Health Services Research and Policy, London School of Hygiene and Tropical Medicine, London, UK; 9 Institute of Social Marketing, University of Stirling, Stirling, UK; 10 Alcohol Focus Scotland, Glasgow, UK

**Keywords:** Alcohol, Premises licensing, Availability, Outlet density, Public health, Local alcohol policy, Natural experiment, Composite measure

## Abstract

**Background:**

Recent regulatory changes in the system by which premises are licensed to sell alcohol, have given health representatives a formal role in the process in England and Scotland. The degree to which local public health teams engage with this process varies by locality in both nations, which have different licensing regimes. This study aims to critically assess the impact on alcohol-related harms - and mechanisms - of public health stakeholders’ engagement in alcohol premises licensing from 2012 to 2018, comparing local areas with differing types and intensities of engagement, and examining practice in Scotland and England.

**Methods:**

The study will recruit 20 local authority areas where public health stakeholders have actively engaged with the alcohol premises licensing system (the ‘intervention’) and match them to a group of 20 lower activity areas using genetic matching. Four work packages are included: (1) Structured interviews and documentary analysis will examine the type and level of intervention activity from 2012 to 2018, creating a novel composite measure of the intensity of such activity and will assess the local licensing system and potential confounding activities over the same period. In-depth interviews with public health, licensing, police and others will explore perceived mechanisms of change, acceptability, and impact. (2) Using longitudinal growth models and time series analyses, the study will evaluate the impact of high and low levels of activity on alcohol-related harms using routine data from baseline 2009 to 2018. (3) Intervention costs, estimated National Health Service cost savings and health gains will be evaluated using the Sheffield Alcohol Policy Model to estimate impact on alcohol consumption and health inequalities. (4) The study will engage public health teams to create a new theory of change for public health involvement in the licensing process using our data. We will share findings with local, national and international stakeholders.

**Discussion:**

This interdisciplinary study examines, for the first time, whether and how public health stakeholders’ involvement in alcohol licensing impacts on alcohol harms. Using mixed methods and drawing on complex systems thinking, it will make an important contribution to an expanding literature evaluating interventions not suited to traditional epidemiological research.

## Background

### Alcohol

Alcohol consumption is a major contributor to the preventable burden of disease in the UK and internationally [1, 2], as well as adverse social outcomes like crime and violence [3–5]. In 2016 there were 7327 alcohol-specific deaths in the UK, an age-standardised rate of 11.7 deaths per 100,000 population [6]. There were 339,000 hospital admissions related to alcohol consumption in 2015/16, an increase of 22% compared to 2005/06 [7] Alcohol harms are socially patterned, making alcohol a key driver, and reflection, of health inequalities [8–14].

### Alcohol Availability & Harm

Systematic reviews, and reviews of reviews, have concluded that legislative measures, including control of the ease with which alcohol can be obtained, can be effective in reducing alcohol-related harms [15–17]. These ‘availability’ interventions include limits on the age at which alcohol may be purchased, as well as controls on the number and proximity of outlets selling alcohol (physical availability) and their hours of sale (temporal availability). There is consistent evidence suggesting an association between increased physical and temporal availability of alcohol and higher rates of consumption and associated alcohol-related harms [18–23], including several UK studies [24–27].

Two recent studies found that local authorities in England with a more intensive alcohol premises licensing regime experienced an additional 5% reduction in alcohol related hospital admissions rates from 2009 to 2015 (or 2% annually) [28] as well as an additional 4–6% reduction in public nuisance and alcohol-related crime rates [29], compared with what would have been expected had these local areas had no active licensing policy in place [30]. The density of alcohol outlets has been shown to be higher in deprived areas in both England and Scotland [25, 31]. Whilst the direction of causation for this relationship is unknown, it raises the possibility that alcohol premises licensing policy could have a greater positive impact on health harms in these areas, and even reduce alcohol-related health inequalities [25, 32].

The extent to which increased availability causes alcohol harms, and if so, the mechanisms by which effects are exerted remains unclear, since much of the research is cross-sectional and the validity of measures of the availability of alcohol premises is variable [21, 33–37]. A recent review of 160 studies found that a causal relationship between public health activities, specific local licensing controls, indicators and types of availability and alcohol-related harms is not clear or consistently demonstrated in the literature [33]. The same study noted the difficulty of translating the research into practice, due both to these limitations and the lack of clear theories of change [33]. Examining the relationship between these three sets of variables – public health team activity, the licensing regime, and local level health/crime outcomes – is the core focus of this study.

### Availability in the UK context

In England and Scotland, in a system similar to that of several other countries, the sale of alcohol requires a licence issued by local government bodies known as licensing committees or boards [21, 32, 36, 38, 39]. In England, there are no statutory restrictions on hours of sale. In Scotland alcohol cannot be sold for ‘off-premises’ consumption (i.e. to take away) outside the hours of 10.00 and 22.00. The hours of sale for a given premises are determined by the conditions of the licence granted by the local licensing committee in both nations, within the statutory restrictions in Scotland.

Historically, the UK licensing system has had a primary focus on limiting public disorder, though health considerations have played a limited part in motivating legislative change [40, 41]. However, legislation passed in 2003 (England and Wales) and 2005 (Scotland) introduced major changes, including the introduction of ‘licensing objectives’ to guide licensing decisions. These objectives are (1) to prevent crime and disorder, (2) to promote public safety, (3) to prevent public nuisance (4) to protect children (and young people) from harm, and, in Scotland but not England, (5) to protect and improve public health [42, 43]. The essential principle of current licensing law in both Scotland and England is the assumption that alcohol licence applications will be approved unless a) there is a representation from a ‘responsible authority’ (‘statutory consultee’ in Scotland) or other party and b) that representation successfully demonstrates that the granting of the licence would undermine one or more of the above licensing objectives. Under the Licensing (Scotland) Act 2005, local National Health Service (NHS) administrations (‘health boards’) in Scotland were designated as statutory consultees; in England and Wales, lead professionals (Directors of Public Health) in each public health team, based within local government, were added as a responsible authority in 2011 under amendments to the 2003 Act.

Licensing authorities in England and Scotland are required to produce a ‘statement of licensing policy’ (SLP), every 5 years in England and normally every 4 years in Scotland. The SLP should outline the authority’s strategic approach to promoting the licensing objectives. In Scotland, such polices must include an ‘overprovision’ statement on the extent to which the whole or any part of the geographic area within their remit is considered ‘overprovided’ with alcohol outlets. A licence application may be refused in Scotland on grounds of overprovision alone. Furthermore, in these areas, the assumption that the application will be approved is reversed, and the onus shifts to applicants to demonstrate why the application will not undermine the licensing objectives. In England and Wales, local authorities can designate specified zones to be ‘cumulative impact areas’ (CIAs), which also reverses the assumption that applications in these areas will be approved.

### Public health engagement in licensing

Following the enhancement of their statutory roles, many professionals with an interest in reducing alcohol-related harms have increased their engagement with premises licensing [44–50]. As well as public health teams in local government in England, in Scotland this work has involved NHS public health departments, NHS professionals with a strategic remit to reduce alcohol-related harms, and professionals based in ‘Alcohol and Drug Partnerships’. Hereafter, for ease of writing, we use the term ‘Public Health Team’ or ‘PHT’ to describe any combination of these groups.

Research in Scotland found that early public health involvement achieved mixed results, with some areas introducing large-scale overprovision policies, and others strongly resisting public health engagement [44, 45, 51]. In England and Wales, PHTs have also faced challenges in adapting to the licensing environment [32, 41, 50, 52]. PH engagement in licensing forms part of a wider, interactional system involving ‘responsible authorities’ (such as fire, police and child protection authorities as well as health bodies), licensing committees, the alcohol trade and, in some cases, the general public. In engaging with this system, and with support from national organisations such as Public Health England (PHE) and Alcohol Focus Scotland (AFS), PHTs have developed a range of approaches [39, 53].

PHTs may, for instance, make representations directly to licensing authorities, provide data in support of a representation by the local police or Trading Standards, respond to consultations on cumulative impact policy or, as has been more common in Scotland, take the lead in developing the case for the establishment of overprovision areas [44, 49, 51]. Some PHTs have developed processes for reviewing and responding to licence applications, collated local datasets on outlet density and alcohol-related harms, supported the development of licensing policies, involved local communities, or directly engaged with licence-holders [46, 47, 49, 51, 54, 55]. These approaches are used to varying degrees of intensity and in varying combinations in local areas across England and Scotland, creating a natural experiment that has yet to be robustly evaluated and which is the focus of the current study.

### Conceptualising public health engagement in licensing within a complex system

In order for public health engagement in licensing to reduce alcohol-related harms, it would need to bring about positive changes in a complex system through which such harms, and related inequalities, arise. The elements of this complex system affect each other in sometimes subtle ways, with changes potentially reverberating throughout the system [56].

Key characteristics of complex systems are emergence, feedback and adaptation [56]. The drinking environment consists of a number of heterogeneous, evolving and interacting components, which exhibit circular causality and emergent properties [57]. Alcohol-related harms can be considered an emergent property of the system of alcohol production, distribution, marketing and sale; and alcohol’s role in a society in a given time and place. A simple example of a feedback loop in the alcohol licensing system could be that a reduction in outlet density in an area could reduce the visibility and convenience of drinking. In turn this could lead to fewer people choosing to drink, which could reduce the demand, and therefore the viability of alcohol retail outlets, potentially further reducing the number of outlets and alcohol availability. Adaptation refers to adjustments in behaviour in response to intervention, such as that remaining alcohol outlets might reduce their prices to try to boost demand; or might increase their prices in response to reduced competition in the market.

In seeking to build understanding of the relationship between local public health engagement in licensing, the nature of the local licensing regime, and alcohol-related harms, it is important to recognise this complexity. The study does not attempt to examine the entire alcohol system, but evaluating this natural experiment in which some PHTs, but not others, actively engage in the alcohol licensing system will require consideration of broad potential mechanisms of impact. These include: (i) how PHT activity might impact directly or indirectly on the licensing system (for example through licensing practitioners, licensees, applicants and other responsible authorities such as the police), and potential feedback loops or adaptations arising from such impact; (ii) how the licensing system might impact on consumption and/or harms (through availability, visibility, pricing policies or quality of premises) and (iii) how other interventions or trends in public health or licensing might influence, add to, or counteract such impacts. In practice, we will explore and measure effects across a range of domains using interrupted time series analyses and extensive qualitative enquiry with diverse actors and documentation sources.

### ExILEnS study aim, research questions & objectives

This study will, for the first time, seek to robustly measure PHTs’ involvement in the alcohol premises licensing system over time, and assess whether greater levels of involvement are associated with reduced alcohol-related harms. Given the complexities referred to above, the study will include a strong focus on processes and mechanisms, as well as assessing health, crime and cost-related impacts.

The aim of this study “Exploring the Impact of alcohol premises Licensing in England and Scotland” (ExILEnS) is:

To critically assess the impact and mechanisms of impact of public health stakeholders’ engagement in alcohol premises licensing on alcohol-related harms in England and Scotland from 2012 to 2018 by comparing areas with differing types and intensities of engagement.

The primary research question is:

1. Does intensive public health engagement in alcohol licensing reduce alcohol-related harms, in local authorities where such activity exists, compared with authorities with low levels of, or no, such activity?

Secondary research questions are:

2) What are the costs and cost-savings, mechanisms of action, and impact on health inequalities of public health engagement in licensing?
3) How do engagement, processes, acceptability, and outcomes vary between Scotland (where a public health objective for licensing exists) and England and from PHTs and licensing perspectives?

This study will contribute to understanding the potential mechanisms of effect of such PHT activity within a complex system and is intended to generate detailed, policy-relevant evidence that can be acted on locally, as well as informing potential national legislative changes and, where appropriate, international licensing regimes.

The study has four sets of objectives addressed by four corresponding work packages. The objectives are illustrated in Table 1.

**Table 1:** Work Packages & Objectives

1. INTERVENTION SCOPING & PROCESS EVALUATION: To describe and explore PUBLIC HEALTH TEAM (PHT) engagement in alcohol premises licensing, the local licensing regime and related processes in 20 high activity and 20 low activity PHTs over the period 2012–2018. a. Identify and recruit 40 local PHTs in England and Scotland that vary demographically and in the timing, breadth, components and intensity of their efforts to engage in alcohol premises licensing since 2012. b. Establish a clear picture of PHT, licensing and confounding activity in each area from 2012 to 2018. c. Establish measurable indicators of the intensity and costs of PHT engagement in licensing and local licensing activity in each area. d. Explore perceived mechanisms of change and real and perceived barriers to PHT engagement in licensing, from the perspectives of public health, licensing, police and other stakeholders.	
2 ALCOHOL HARMS EVALUATION: To quantitatively evaluate whether PHT engagement in licensing has a measureable impact on health harms and crime rates using routine data from 2009 to 2018. a. Match the selected intervention local areas to 20 best possible control areas using genetic matching. b. Collect quantitative data on a set of key alcohol harm and crime outcome indicators on which subsequent evaluation will be based. c. Evaluate if, and to what extent, the intensity and components of the intervention are associated with subsequent measureable changes in the key outcome indicators.	
3. WIDER IMPACTS, COSTS AND DISTRIBUTION OF EFFECTS: To examine implementation costs, estimate the short-term impact of PHT engagement in licensing on alcohol consumption and the longer-term impact (up to 20 years) of the intervention on health and healthcare costs and explore the likely distribution of effects across the population. a. Estimate and compare the overall costs to PHTs of implementation activity b. Develop locally-specific policy models for each active intervention area. c. Use these models to estimate the wider impacts of the intervention in terms of long-term health benefits, NHS cost savings and how these impacts may affect health inequalities d. Estimate the potential impact of high intensity PHT activity in two exemplar areas (one in England, one in Scotland) which are not currently active.	
4. IMPACT OF FINDINGS: a. Revise and refine hypothesised theories of change to qualitatively examine how PHT activities and key aspects of the licensing system may lead to changes in licensing outcomes and related harms. b. Synthesise all findings, plan dissemination and identify recommendations for practice, policy and future research and disseminate.	

## Methods/Design

### Overview

The study will employ a mixed-methods natural experiment design with four Work Packages (WPs):

1. Mapping and exploring public health engagement in the alcohol premises licensing system, and the local licensing system in place, from 2012 to 2018; in 40 local authority areas in England and Scotland, using documentary analysis, semi-structured interviews and in-depth interviews.
2. Evaluating the impact of high and low levels of public health engagement on alcohol harms using longitudinal growth models and time series analyses using routine data from 2009 to 2018.
3. Using the Sheffield Alcohol Policy Model (SAPM) [58–60] to evaluate intervention costs, estimated NHS cost savings, and health gains and to estimate impact on alcohol consumption and potential impact on health inequalities (gender/socioeconomic).
4. Synthesising and analysing findings from all WPs, with input from public health and licensing stakeholders, to create a description of the alcohol licensing system in local authorities in England and Scotland, and a theory of change relating to the impact of public health engagement in that system and the influence of licensing on alcohol harms.

### Service users/public involvement

Two members of the public have been recruited to support the study advisory group and will be supported to attend and input to those meetings. TN, formerly Head of Regulatory Services for Brighton Council is a co-investigator on the study. The UK Centre for Tobacco and Alcohol Studies hosts a public involvement group ‘the alcohol discussion group’ which meets 2–3 times a year and which has reviewed the study including contributing to its published lay summary.

### Recruitment and allocation

#### Intervention areas

The ‘intervention’ in our study is defined as the presence of a PHT that has been active across multiple aspects of public health engagement in alcohol premises licensing. The unit of analysis for each selected area will be a lower-tier local authority area in England or a single licensing board area in Scotland.

We will recruit 20 PHTs who have been actively seeking to influence alcohol licensing in at least one local authority/licensing board area under their remit. Where a PHT is active in several local areas, the one in which they deem themselves to be most active will form the ‘intervention’ area for the study. The study will also identify 20 ‘control’ local authority areas where little or no PHT involvement in licensing has occurred. Six intervention and matched control areas in Scotland will be included along with 14 intervention and matched control areas in England, giving 40 areas in total.

All PHTs were informed about the proposed study by PHE and AFS prior to funding being secured and were invited to express interest in being involved (44 areas expressed interest at that stage). We will issue further calls for expressions of interest by email, promote the study through events organised by PHE and AFS and via relevant other organisations and events such as the Institute of Alcohol Studies, the National Licensing and Public Health Network, and invite participation through direct contacts with local areas where members of the team and colleagues have previously worked. We will build on all of these networks to publicise the opportunity to participate in the study.

From those areas that have expressed interest, we will select active intervention areas based on a combination of the following:

- advice from expert bodies including our study advisory group;
- published reports and case studies [44, 47, 61–63]
- publically available information on involvement in other licensing initiatives (such as the home office initiative ‘local alcohol action areas’ [64, 65]);
- prior research by members of the team and colleagues e.g. [49, 51, 66]
- scoping calls with local areas to clarify levels of activity since 2012 and continued interest in participating in the research.

Selection will primarily focus on those areas with sustained high intensity public health team engagement in licensing from the earliest time point, but will also aim to include at least one local area from each region in England (Northeast & Yorkshire; Northwest; Midlands & East England; London & the Southeast; Southwest); and both urban and rural areas. All local authorities in England and Scotland will be eligible for inclusion with the exception of the three Scottish island authorities due to the relatively low number of licence applications under consideration.

#### Control areas

In England, there are 326 lower-tier local authority areas in total and potential control areas will be identified using genetic matching. In a natural experiment such as this, it is not feasible to randomise PHTs to active or inactive groups, but it is important to address potential bias that may have been introduced by pre-existing differences between intervention and control areas. Genetic matching uses algorithms to select control areas to ensure that intervention and control areas are as similar as possible on predetermined covariates (Table 2). It seeks to minimise the Generalised Mahalanobis Distance (GMD), which is here a weighted multivariable indicator of the difference between intervention and control areas across all chosen covariates.

We will use genetic matching to identify 14 control areas for the 14 English intervention areas. Following matching, we will attempt to recruit the control areas with the most similar distributions of matching variables to the intervention areas. If an identified control area declines to participate, or is unsuitable due to high activity levels, we will exclude this area and re-run the matching with the remaining potential control areas.

Due to the much restricted pool of potential control areas in Scotland, compared to England, the same matching method could not be used. Instead, the values of every covariate outlined in Table 2 were normalised across all local authorities such that the highest value corresponded to 100 and the lowest to 0. For each of the 6 selected case areas, the cumulative root mean square error across all covariates was calculated for every potential control. The final set of control areas was identified by selecting the pool of controls which minimised the cumulative error across all 6 case-control pairs. Where a potential control area from this pool declines to participate in the study or is found to be a high activity area, we will select the next best alternative using the same cumulative error-minimisation approach.

**Table 2:** Selected covariates for genetic matching

Variable category	Country	
	England	Scotland
Deprivation/inequality	-Percentage of population living in a rural area	-Percentage of population living in a rural area
	-Percentage of population living in area in most deprived quintile -Long-term unemployment (jobseekers claimant > 12 months)	-Scottish index of Multiple Deprivation score (average score across data zones for each local authority)
Population/outlet density	-Population density per square kilometre	-Estimated mid-year population -Population density per square kilometre -On-licence density -Off-licence density
	-On-licence density	
	-Off-licence density	
Alcohol-related harm	-Alcohol-related hospital admissions (standardised rate; narrow measure) -Alcohol-related violent crime	-Alcohol-related hospital admissions (standardised rate)
Demographic variables	-Median age	-Median age

Potential variables for inclusion in the matching processes were identified using a modified Delphi approach [67]. Matching variable selection was refined following an evaluation of covariate balance from initial propensity score matching analyses (presented at ExILEnS team meeting 22.09.17), and discussion with members of the ExILEnS team (de Vocht, Angus, Egan and Maani-Hessari). All of the covariates included in the matching process were measured at baseline (2009–2011).

Table 2 lists the final covariate set which will be used for matching for English and Scottish local authority areas. The covariate list differed between the two countries due to differences in the publicly available data at local authority level.

### Outcomes of interest

Longitudinal data on a set of key alcohol harm outcome indicators’ will be collected for each intervention and control area from 2009 to 2018 as shown in Table 3. Some alcohol-related harm takes time to develop so there will be some lag. Implementation of these lags will be specified prior to the analyses being undertaken by reference to relevant literature and in consultation with our advisory group.

**Table 3:** Outcome Indicators & Data Sources

Outcome indicator	Source	
	England	Scotland
Quarterly alcohol-related hospital admissions	PHE	Information Services Division (ISD) Scotland
Quarterly alcohol-related and alcohol-specific mortality	PHE	National Records of Scotland (NRS)
Quarterly reported crime rates with significant attribution of alcohol abuse (violent, sexual, and public order offences)	Office for National Statistics (ONS)	Scottish Government
Weekly ambulance call outs for weekdays/weekends, both daytime and night-time	English Ambulance Services	Scottish Ambulance Service
Weekly A&E attendance rates for weekdays/weekends, both daytime and night-time	NHS Hospital Episode Statistics (HES) Data Access Request Service (DARS)	ISD Scotland

### Power calculations

No quantitative data is available on the effect of public health engagement in licensing on alcohol-related harms. The assumed mechanism by which such engagement might reduce harms however, is by influencing the local licensing system. Statistical power estimations therefore have been based on effect sizes from two recent studies of the effect of alcohol premises licensing on alcohol-related hospital admissions and reported crime rates at the level of ‘lower tier local authority’ (LTLA) in England [28, 29]. Areas with active alcohol licensing policies had an average additional 2% (95% CI -3%:-2%) annual reduction in alcohol-related hospital admissions in the period up to and including 2013 compared to those without such policies [28]. Similarly, for the period up to 2013, an additional 4–6% annual decrease was seen in alcohol-related violent crimes, sexual crimes and public order offences in areas with active licensing policies compared to those with none [29].

We used the methodology developed by Edland for power calculations of linear mixed effects models with random slope [68]. Based on the previous studies, we conducted separate sample size calculations for alcohol-related hospital admissions and reported crime rates. For both analyses we assumed a standard level of statistical significance α (5%) and statistical power β (80%), and further assumed a 9-year follow-up (2009–2018) and a two-sided alternative. Table 4 outlines the detectable effect size with 20 intervention and 20 control areas.

**Table 4:** Sample size data

	Expected average effect size %/year (slope)	Between-slope variance	Residual variance model	Number of areas in each group
*From previous study of impact of licensing on alcohol-related crime* [29]				
Crime rates^a^	−4.00% (−0.04)	0.003	0.03	29
	−5.00% (−0.04)	0.003	0.03	19
	−6.00% (−0.06)	0.003	0.03	13
*From previous study of impact of licensing on alcohol-related hospital admissions* [28]				
Rates	−2.31% (−0.229)	0.110	0.011	34
*Current study - minimum detectable effect size with proposed sample size*				
20 areas per group	−3.00% (−0.296)	0.110	0.011	20

^a^Effect size is a range between 4 and 6% as estimated by d Vocht et al. (2016) using quadratic trends

Based on Table 4, we expect the study to be able to detect effects on our outcomes within the range found in previous observational studies with 20 intervention and 20 control areas.

Previous studies have not evaluated the impact of local licensing on attendances at Accident and Emergency (A&E) departments. Injuries and accidents are the largest single driver of A&E attendances [69] and are strongly linked to acute alcohol consumption and intoxication [70]. Therefore we expect an effect size in or around the range found for crimes as these are also strongly linked to acute alcohol consumption [71].

### Data collection

We will develop a series of data collection tools for use in each intervention and control area and keep detailed records of what data is collected and how it is obtained. Data collection will be completed in the 20 intervention areas via sourcing and analysis of relevant documentation by email; an initial site visit, and structured telephone interviews with public health and licensing practitioners. Drawing on the research team’s (which includes practitioner advisors with expertise in public health and licensing) knowledge, we have drawn up an initial list of documents to request. The document request will be made by email prior to each site visit so that fieldworkers can extract relevant data from the documents to populate a series of timelines. These timelines will describe different types of activities relevant to PHT engagement in licensing at six monthly intervals over the study period. The timelines will form the dataset upon which we will apply our measure of intensity of activity. The initial list of activities that data collection will focus on is provided in Table 5 below.

**Table 5:** List of local Public Health and Licensing activities that data will be collected on

Intervention Components (Indicators)	Licensing activity/regime
a. A systematic process for review of new licensing applications & variations (known point of contact, clear criteria, use of routine data) b. Active response to applications (liaison with responsible authorities, licensing reps, applicants; representations) c. Development of bespoke datasets (robust/ systematised local data collection on harms etc.) d. Engagement with licensing authorities (meetings, awareness raising, licensing policy input) e. Activity towards development of cumulative impact/overprovision areas (submissions, representations, consultation) f. Public health-led activity to involve the public/local communities (depth, breadth of involvement, activity of local licensing fora) g. Public health-led engagement with licensees (‘Reducing the Strength’ schemes; advertising/ promotion bans) h. Any other public health led activity to influence licensing/licensees.	i. Licence application levels, types, conditions j. Licence decisions k. Cumulative impact/overprovision policies/areas l. Outlet density by type. m. Late night levies n. Health commitment in licensing policies o. Reducing the strength scheme sign up p. Local advertising/promotion ban q. Health as a licensing objective (if introduced locally in England) r. Any other relevant elements

Most of these indicators and interim licensing outcomes leave a documentary trail (e.g. databases, policy statements, records of meetings) which we will identify with assistance from local contacts and supplement with further information obtained from interviews. The list will be further developed following the initial scoping calls with potential intervention areas and consultation with the study advisory group, informed by prior research. The development process will also be informed by discussion with the lead authors of studies which developed three other alcohol policy measures in the literature [72–74].

The intervention area site visits will provide fieldworkers with the opportunity to interview alcohol leads from the local PHTs and to meet licensing representatives. The main purpose will be to fact-check and add to the various activity timelines produced from the document analysis, and to request further documents if necessary. Besides interviewing current alcohol leads, fieldworkers will (if necessary) seek interviews with previous leads to ensure data collection covers the whole study period. The availability of interviewees with knowledge of the whole study period will be one of the criteria considered when selecting participating areas. The data collection will be completed by sourcing relevant documentation by email and via further structured telephone interviews as needed. Data collection in control areas will involve similar documentation analysis and structured telephone interviews. It is anticipated that the type and quantity of documentation will vary greatly by area, as some intervention areas will have engaged in more relevant activities than others, whilst control areas will be selected because of their lack of engagement.

We will also collect data on potential confounding activities in each area such as: local initiatives around alcohol screening/brief advice; public information & education initiatives; police-led initiatives in the night-time economy; drink driving initiatives; industry-led best practice schemes; or any other major relevant confounding activity. Whilst we will aim to map confounding activities on our timeline we anticipate that some confounders are likely to be specific to particular area contexts and knowledge of them will emerge during the course of our fieldwork. We will incorporate analysis of wider confounding factors into our overall analysis and ensure that it is taken into account of when interpreting our findings, in particular how PHT activity in the licensing arena is positioned within the wider system of factors affecting licensing practices and alcohol harms.

Intensity of PHT and licensing activity will be assessed by two separate composite ordered categorical measures which will generate an intensity score for PHT activity and for licensing activity within each area in a given period. Firstly, all data will be analysed using NVivo. Then the measures will each be devised in an iterative process of development and testing using analysed data and consultation with UK and international experts.

The first draft of each measure will define dimensions and indicators for different categories of PHT or licensing activity and will be developed based on current research literature and published best practice guidance. This draft will be used to code data a subset of interventions areas, and then all intervention areas, revising as needed at each stage. The resulting measure will be sent to UK-based public health and licensing experts, to review for clarity, completeness and relevance and then further revised based on their feedback. The range of practice for each included indicator of intensity will be analysed by each researcher, and discussed and combined to develop a single measure and defined grading scales for each included indicator. This version will then be applied independently by two researchers to a subset of intervention and control area data, and the resulting scores compared in discussion, checking for consistency and face validity. The final stage of development will involve deciding, using our data, and in consultation with experts, whether and how different dimensions or indicators within the measure should be weighted. Weightings will also be informed by parallel work by the team to further develop a theory of change for PHT involvement in licensing.

Once the measures have been finalised using this iterative process, they will be reapplied to the data for all intervention and control areas to calculate intensity scores for each 6 month period April 2012 to March 2019. Two researchers will apply the measure to 10% of areas (*n* = 4) to examine inter-rater reliability. Where identified, discrepancies will be discussed and assessed by a third reviewer as needed. The scoring system will then be revised and another 4 areas assessed by two reviewers. In the event of further serious discrepancies, this process will be repeated. We will report the inter-rater reliability of the finalised measure.

The agreed intensity scores will be used to examine the relationship between intervention intensity, licensing regime activity and alcohol-harm outcomes.

The final element of primary data collection will involve in-depth qualitative interviews with public health practitioners and other stakeholders in each intervention areas (up to 80 in total). These other stakeholders may include: local authority licensing practitioners, police, trading standards officers, licensing board members and others. These interviews will focus on alternative perspectives on public health involvement in licensing. Semi-structured topic guides will be developed in consultation with our practitioner representative and advisory group in line with our research questions, informed by relevant literature. All interviews will be audio-recorded. Audio-recordings will be transcribed verbatim by experienced transcribers, transcripts checked for accuracy, and anonymised. Detailed field notes will supplement interviews conducted during site visits to intervention areas and will inform later analysis. Analysis will use a collaborative, qualitative framework approach [75, 76] to identify the themes arising and to compare between England and Scotland, different stakeholders and PHTs.

### Analysis of primary outcomes

We will evaluate temporal trends in all key outcomes from 2009 to 2018 and compare these in intervention and control areas using hierarchical log-rate growth models. This method was previously used by members of the research team to investigate the association between a metric of ‘licensing activity’ and alcohol related hospital admissions [28, 30] and crime rates [29] in England.

An additional feature of this study over and above those studies, is the chunking of the intervention intensity measure into 6-month intervals enabling specific exploration of causal effects through inclusion of pre/post indicators and interactions in the growth models as well as the use of ‘Differences-in-Differences’ statistical methods [77]. Inferences about causality can be made through quantitatively evaluating, using a pre-specified plan based on the emergent theory of change, whether there is statistical evidence of changes in longitudinal trends in outcome measures that coincide with the expected effect of the intervention (and is not present in the corresponding control area). Where data is available we will analyse outcomes by deprivation and gender.

### Cost-effectiveness analysis and modelled impacts

Within the primary data collection, estimates of staff time and resource use involved in PHT intervention activities will be obtained for each intervention area. We will use this data to estimate the cost of PHT engagement in licensing, both overall and in terms of individual components of activity.

The Sheffield Alcohol Policy Model (SAPM) is an advanced epidemiological simulation model which has previously been used to estimate the impacts on alcohol consumption and related harms of a wide range of alcohol policies, including those affecting price, outlet density and licensing hours in both England and Scotland at the national level [58, 78, 79]. Previous versions of SAPM have been developed at national level. Within this study we will develop new, Local Authority-level models for each intervention area using local data on alcohol consumption, demography and alcohol-related harms.

Using these local versions of SAPM, we will produce estimates of the changes in alcohol consumption, alcohol-related harm and associated healthcare costs, and the distribution of these changes across the local population, resulting from the implementation of local policies and interventions. We will also create models for 2 exemplar control areas, selected from the control areas, based on data availability and the extent to which the PHT is interested in increasing the intensity of their activity. These models will produce estimates of the potential impacts of increasing PHT activity on health and crime outcomes in these areas.

As described above, PHT engagement in licensing is intended to affect the local alcohol licensing system, and therefore (assuming licensing policy directly affects alcohol consumption and drinking behaviour) to impact on key outcomes measured in the general public including health and crime. However, alcohol consumption and harms are not evenly distributed across the population, and intervention activity may impact differently on different population groups. Considering this variation is key to understanding both the true impact of an intervention and also the potential for the intervention to alter these distributions and narrow or widen existing socioeconomic and gender inequalities in health [80–82]. SAPM addresses this by modelling baseline consumption and harm, policy effects, and all outcomes fully stratified by deprivation as well as age, gender and drinking level.

The study will explore these issues and the potential for licensing engagement and policy to affect these socioeconomic gradients through: a) using the Local Authority versions of SAPM and b) exploring the differential impact of the intervention on health outcomes by gender and socioeconomic group (defined by quintiles of the relevant Index of Multiple Deprivation or other relevant markers) to establish the potential of intervention activity to reduce (or exacerbate) the substantial existing inequalities in alcohol-related harms [12].

### Impact

**Fig. 1:**
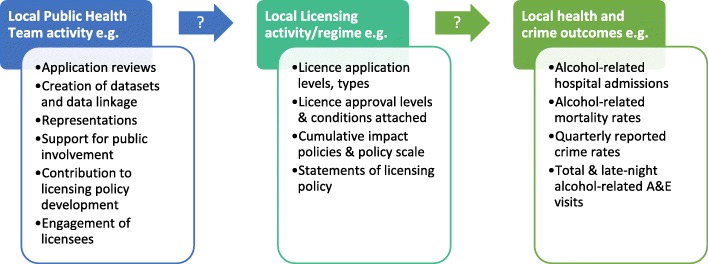
Simplified theory of change

**Fig. 2:**
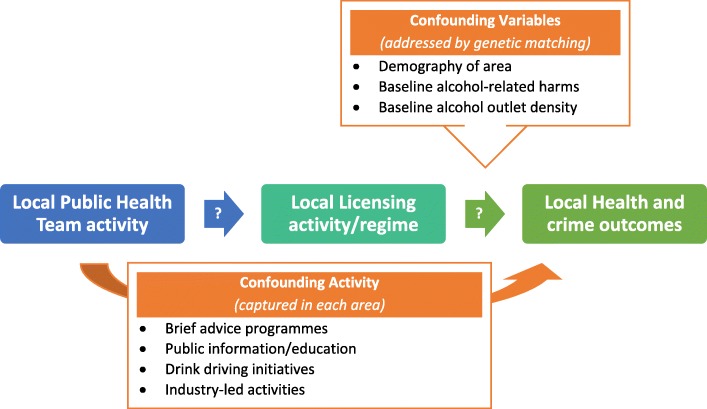
Simplified theory of change with major confounding variables and activity added

Prior to the study’s commencement we developed a simple linear theory of change to guide our evaluation design. This can be seen in Fig. 1, in which different aspects of local PHT activity are theorised to contribute to changes to local alcohol licensing regimes, which in turn impacts on health and crimes outcome. Figure 2 adds some confounding factors to the theory. Using data collected during work package 1 we will revise and expand on this simple theory of change, producing a fuller list of activities, confounders and incorporating a systems lens.

In developing the revised theory we will consider multiple mechanisms of action, some of which may be complementary and some competing, as well as feedback loops and adaptations in the licensing system resulting from different approaches to public health involvement. For example, some actions may focus on indirect mechanisms of action (influencing the nature and type of applications accepted e.g. licensed arts venues but not nightclubs [49]) whilst others may focus more directly on reducing the number of new licences granted through stronger licensing policy.

The emergent theory of change will be informed by practitioners’ views expressed in interviews, and detailed information on local licensing policy and decisions gathered via documentation analysis from WP 1 as well as outcome data from WP 2. The final theory (or theories) will be developed by the full study team, in consultation with the study advisory group and practitioners via a ‘stakeholder workshop’. We will invite all participating areas to attend this workshop at which we will present emerging findings and the draft theory of change.

### Dissemination

We will draw on our close links with PHE, AFS, the Alcohol Health Alliance, Alcohol Research UK, Cancer Research UK and others to ensure that our findings can influence local public health practice and national advocacy work. PHE host a National Public Health and Licensing Network (co-chaired by co-investigator Nicholls), whereas AFS host regular knowledge exchange events in Scotland for local teams, runs annual licensing conferences, and publishes a monthly e-newsletter. All of these organisations will disseminate study information and findings through established mechanisms and will guide us on appropriate formats for each audience. Our findings will also influence teaching and capacity building through annual alcohol policy courses delivered on behalf of the UK Centre for Tobacco and Alcohol Studies.

We expect to publish several peer-reviewed journal papers from each work package and will disseminate findings at UK and international conferences.

## Discussion

This study is important because alcohol is a major cause of health and social harms and regulation of its availability is a long-established mechanism for reducing those harms. Given the public health imperative to improve population health and wellbeing, there is a clear case for research designed to better understand how policy levers currently in place to influence alcohol availability are utilised by public health teams and what their impact may be. This research will help address acknowledged areas of uncertainty around whether and how current approaches to the regulation of alcohol availability are beneficial.

This natural experiment builds on the methodology and findings of recent work demonstrating an impact of licensing on health and crime outcomes [21, 28, 29], and will examine for the first time the effect, and mechanism of effect, of public health involvement in licensing. Using an interdisciplinary mixed-methods approach, and drawing on complex systems thinking, it will take into account the complexity of the relationship between public health activity, licensing decisions, and alcohol harms, as recommended in recent reviews [33–35]. It will involve in-depth examination of practice, acceptability and feasibility across two jurisdictions (England and Scotland) to build on earlier work [41, 44, 49, 51, 55, 66, 83].

The wide range of current public health practice will allow this study to generate qualitative contextualised data on the challenges and opportunities for PHTs seeking to affect alcohol-related harms through engagement with local premises licensing and will examine theories of change. At a local level, the study will be able to examine how public health teams can tailor their approaches to the licensing system to their local context, to maximise their likelihood of success, based on a clear theory of change.

As public health engagement in this area is potentially resource-intensive, findings from ExILEns will inform decisions on about whether this activity represents the best use of time for public health teams. If it is shown that there are measurable benefits, this will help to make the case for greater investment in public health capacity and/or potentially greater legislative support through the introduction of a public health objective in England. If, however, our findings suggest limited effects of public health activity in this arena, or little potential for licensing policy to materially affect alcohol-related harms, then this will contribute to current debate around more substantial legislative changes [84]. Should this study have null findings, this may, therefore, be as significant in policy terms as a demonstration of positive effects.

## Abbreviations

A&E: Accident and Emergency
AFS: Alcohol Focus Scotland
CIA: Cumulative impact area
DARS: Data Access Request Service
ExILEnS: Exploring the impact of licensing in England and Scotland
GMD: Generalised Mahalanobis Distance
HES: Hospital Episode Statistics
ISD: Information Services Division
LTLA: Lower tier local authority
NHS: National Health Service
NRS: National Records of Scotland
ONS: Office for National Statistics
PH: Public Health
PHE: Public Health England
PHT: Public health team
SAPM: Sheffield Alcohol Policy Model
SLP: Statement of licensing policy
WP: Work Package
